# Comparative study of sexual health knowledge and practices among sexually active adolescent girls in co-educational and girls-only secondary school in Calabar, Nigeria

**DOI:** 10.11604/pamj.2022.43.157.35942

**Published:** 2022-11-25

**Authors:** Nkese Mkpanam, Enagu Mpama, Nnette Ekpenyong, Ogban Omoronyia, Iwasam Agbor

**Affiliations:** 1 Department of Community Medicine, University of Calabar, Calabar, Nigeria

**Keywords:** Sexual health knowledge, sexual behavior, adolescent girls, Calabar, Nigeria

## Abstract

**Introduction:**

there is little or no progress towards the attainment of sexual and reproductive health (SRH) targets of the Sustainable Development Goals (SDGs) in many developing country settings. Key SRH gap in these settings includes suboptimal knowledge-based safe sexual practices, especially among adolescent girls as a vulnerable subpopulation. Unique features of school environmental settings including gender segregation have not been harnessed for cost-effective sexual health education, perhaps due to the current paucity of literature. This study was aimed at comparing sexual health knowledge and practices, between sexually active adolescent girls in co-educational and girl-only secondary schools in Calabar, Nigeria.

**Methods:**

cross-sectional comparative study design was used. Sexually active adolescent girls were randomly recruited from co-educational and girl-only secondary schools in Calabar, Nigeria. Validated questionnaire developed by the United Nations Educational Scientific and Cultural Organization (UNESCO), was used to assess sexual health knowledge and behavioral practice. Factors associated with a satisfactory level of knowledge were assessed using the Chi-square test. Data analysis was done using SPSS version 24.0, with the p-value set at 0.05. Ethical approval was obtained before data collection.

**Results:**

one hundred and twenty respondents were studied, comprising an equal proportion of sixty (60) in co-educational and girl-only schools. Mean age and age at sexual debut were 16.4 ± 1.8 and 14.3 ± 2.2 years, respectively. Compared with group 1 (co-education), respondents in group 2 (girl-only) had significantly higher mean knowledge scores (26.1 vs. 30.4, p<0.05). Fifty respondents (41.7%) had a satisfactory level of knowledge of sexual health. There was no significant difference in mean practice scores comparing groups 1 and 2 (20.4 vs. 21.5, p>0.05). Internet use, unmarried parental status, and not living with both parents, were associated with unsatisfactory levels of knowledge on sexual health.

**Conclusion:**

compared with co-educational schools, girl-only schools have better sexual health knowledge, but a similar level of behavioral practices. There is a need for improvement in sexual health education efforts among adolescent girls, perhaps with more focus on coed schools, within the context of potential inherent disadvantage in the school environmental setting.

## Introduction

Adolescence is a critical period of rapid pubertal development, characterized by formation and eventual establishment of sexual behaviors. In many developing countries, due to suboptimal mainstreaming of gender, as well as sexual and reproductive rights of women, adolescent girls constitute key vulnerable subpopulation with largely unmet sexual health (SH) needs [1]. Compared with other global regions, sub-Saharan Africa lags behind in attainment of sexual health targets of the Sustainable Development Goals (SDGs) [2,3]. In Nigeria, several studies in diverse settings have reported high prevalence of non-use of condom, multiple sexual partners and other unsafe sexual practices among in-school adolescent girls [4-7]. For instance, previous study among in-school adolescent girls in the Niger-Delta region, reported non-use of condom in their first and last sexual intercourse among 68.7% and 41.8% of respondents, respectively [4]. There is therefore largely unmet sexual health needs including inadequate support for knowledge-based sexual behavioral practices, amidst rapidly increasing peer-led and media-driven drivers of unsafe sexual practices [8-10]. Besides home or family environment, schools represent vital settings which contribute to sexual behavior, especially through acquisition of correct and incorrect information related to sexual health. In particular, secondary schools have been identified as important settings, for peer-led and/or teacher-driven of sexual health education [11,12]. Yet, the role of school environment on sexual health behavior, may be dependent on gender-based segregation of the structure into single-sex or coed or coeducational schools [13]. Compared with coed schools, rationale for single-sex schools, have been based on perceived better academic and career success, perhaps due to less distractions from opposite sex [13-15]. Single-sex schooling has also been noted to influence attitude towards gender equality, which may in turn determine sexual health behavior [14,15]. Notably, there are physiological and psychosocial differences in the ways that boys and girls process and learn academic as well as non-academic issues [16,17]. Through peer interaction and acculturation, there may also be differences in mode of learning and eventual level of sexual health knowledge comparing girls in coed and girl-only school settings [16-18].

However, the extent to which school settings may influence sexual health knowledge and behavior have been largely under-researched. Paucity of literature includes better understanding of the differential effects of gender-based nature of school environment on the psychosexual relationship with the opposite gender, as well as knowledge-based sexual behavior [19,20]. Also, several arguments for and against single-sex schooling have been based on studies conducted in more developed countries [15,21]. Unfortunately, there is a paucity of literature required for cost-effective evidence-based application of unique features of school environment on meeting sexual health needs of adolescent girls. Previous study in Ibadan, South West Nigeria, found no significant difference in sexual health knowledge difference, though both boys and girls constituted study population, without emphasis on effects of single-sex school environment [22]. Idowu *et al*., in their similar study in coeducational school setting, found 5 times increased odds of risky sexual behavioural practices among in-school adolescents in unsteady sexual relationships [4]. Unfortunately, there is lack of similar studies in single-sex or girl-only schools, therefore limiting evidence-based comparison for assessment of role of gender composition on sexual health knowledge and practices among in-school adolescents. For instance, recent large-scale national surveys in Nigeria, which identified role of community structure on timing of sexual debut and eventual sexual behaviour, excluded assessment of gender-based characteristics of school environment, as a potential determinant of sexual behaviour [23]. Therefore, there is need for better understanding of the role and contribution gender-based composition of school environmental settings, on level of knowledge-based sexual practices, especially among girls as more vulnerable groups [19,20]. This study was therefore aimed at assessing and comparing level of sexual health knowledge and practice among adolescent girls in a developing country co-educational and girl-only school settings. Findings may be useful for improvement in best practices for potential restructuring of school environmental settings towards optimum attainment of knowledge-based safer sexual practices in resource-poor settings.

## Methods

**Study design, setting and population:** comparative cross-sectional descriptive study design was employed in this school-based study among adolescent girls in Calabar, Nigeria. Calabar is a metropolitan city in Southern Niger-Delta region of Nigeria. There are 5 and 47 girl-only and coed secondary schools in Calabar. Like in most other cities in Nigeria, there is high and rapidly increasing population of young people, with adolescents constituting over 40% of census estimates, with male: female ratio of 1: 0.93 [24]. Study population comprised sexually active adolescent girls attending public secondary schools.

**Sample size estimation:** minimum sample size of one hundred and twenty (120), comprising sixty (60) for each group, was determined using the Leslie Kish formula [25].


$$N=\frac{Z^{2}\times P(1-p)}{d^{2}}$$

Where, N is minimum sample size, Z is the standard normal deviate for 95% confidence interval which is 1.96, P is proportion in the target population [which is 96%, representing proportion that were knowledgeable of HIV and its means of transmission as key sexually transmitted infection (STI) in previous study among in-school adolescent girls in Agbor, Southern Nigeria] [26] and d is degree of accuracy which was assumed to be 5%.

**Sampling method and eligibility:** multistage sampling was employed to recruit respondents into the study. In the first stage of sampling, simple random technique by balloting, was used to select one school each, from the list of existing girl-only and coed schools in Calabar. Multi-stage sampling method was used. In the second stage, simple random technique by balloting was used to recruit sixty (60) respondents, from derived sampling frame list of sexually active girls in senior secondary classes, in each of the selected schools. Sexual activity status was determined via respondent´s self-reported, upon enquiry by the student´s most amiable and confidential female school teaching or non-teaching staff in the school. These apparently friendlier staff were determined by the students, during prior interaction with the research team. During enquiry by the chosen staff, only students who could remember or express their experience at sexual debut and last heterosexual sexual intercourse were considered sexually active and eligible to participate. Students who were older than 19 years, as well as those who were admitted to the school within one year, were excluded from the study. Two students, comprising one from each school, were excluded due to illness during the study period.

**Data collection and instruments:** interviewer administered structured questionnaire was used to obtain quantitative data on sexual health knowledge and behavioral practice. Research assistants who were female graduates, received 2-day, 2-hour daily training by the research team and professional counselors on the questionnaire administration including communication with adolescent girls. They were then paid and engaged for data collection, cleaning and entry for analysis by statistician. Data collection was conducted for three (3) weeks beginning on 24^th^ January through 11^th^ February, 2022. The UNESCO-validate questionnaire [27] was pretested, with attainment of Cronbach alpha coefficient of 0.78 before use for data collection. The questionnaire had three sections comprising sociodemographic data (section 1), 62-item section 2 assessing SH knowledge of puberty changes (5), types (10), modes of transmission (15), symptoms (10), complications (6) and prevention (11) of STI and unwanted pregnancy (5).

**Data analysis:** it was aimed at computing and comparing sexual health knowledge and practice scores between the two study groups, as well as assessing factors associated with satisfactory level of knowledge. The responses to knowledge questions were coded as categorical variables. Correct response to each question contributed one unit to total knowledge score as numerical variable. Satisfactory level of knowledge was set at 50% score as benchmark. Section 3, which assessed sexual behavioral practice, had 7-items with 5-point Likert scale ranging from ‘always´ to ‘never´ as categorical variables. This Likert scaling corresponds to score of 5, 4, 3, 2 and 1 for always, frequently, sometimes, occasionally, rarely and never, respectively. Scores were summed up for each respondents as numerical variable. Higher scores indicated safer sexual practices, with minimum and maximum scores of 7 and 35, respectively. The items in section 3 comprised condom use frequency (item 1), condom use insistence when partner differs (item 2), sex under influence of substance (item 3), casual sexual intercourse (item 4), sex before discussing risk factors (item 5), emergency contraception use following unprotected sex (item 6) and exposure to pornography (item 7). Questions indicating unsafe sexual practice comprising items 3, 4, 5 and 7, were reverse-scored. Data analysis was done using SPSS version 24.0. Sexual health knowledge and practice scores were normally distributed, with comparison of mean scores of sexual health knowledge and behavioral practice between study groups using independent t-test. Chi-square test was used to compare proportion of respondents with satisfactory level of knowledge between the study groups. P-value was set at 0.05. Results were presented using tables and cross-tables.

**Ethical consideration:** ethical approval was obtained from the Cross River State Research Ethics Committee before conducting the study (approval number: CRSMOH/RP/REC/2022/228). Informed and written consent was obtained from parents/guardians, while assent was obtained from respondents before data collection. Privacy, confidentiality and voluntary participation were communicated to respondents and adhered to throughout the study. There were no monetary rewards for participation in the study.

## Results

One hundred and twenty respondents were studied, comprising an equal proportion of sixty (60) in co-educational and girl-only schools. Mean age was 16.4 ± 1.8 years, ranging from 14 to 19 years. Mean age at sexual debut was 14.3 ± 2.2 years, ranging from 10 to 18 years. Most respondents were younger than 18 years (77.5%, in Senior Secondary Class 3 (52.5%), and did not live with both parents (59.8%) (Table 1). Half of respondents each, had parents that were unmarried (50.0%) and had sexual debut when they were between 15-17 years old. Efik (26.7%) and Ejagham (23.3%) were the common ethnic groups. Significantly higher proportion of respondents in coed schools had internet as source of information, while teacher-led and other formal teachings was commoner source for girl-only school (p<0.05). There was no significant difference in proportion for other sociodemographic variables comparing the study groups (p>0.05). Result in Table 2 shows level of knowledge of sexual health among respondents in the two study groups. Knowledge of the various components of sexual health was measured on a 62-point scale. Compared with respondents in group 1 (co-educational school), those in group 2 had significantly greater mean knowledge scores overall (26.1 vs. 30.4, p<0.05), as well as for most components of sexual health knowledge assessed, including anatomical and physiological changes at puberty, symptoms, complications and prevention of STI and unintended pregnancy and its consequences (p<0.05). There was no significant difference in mean practice scores comparing groups 1 and 2 for overall scores (20.4 vs. 21.5) as well as scores for each of the seven sexual practice items assessed (Table 3, p>0.05). Fifty (50) respondents (41.7%) had a satisfactory level of knowledge on sexual health, with a significantly higher proportion among those in girl-only compared with co-educational schools (53.3% vs. 30.0%, p<0.05, Table 4). In Table 5 shows results of factors associated with level of knowledge on sexual health. Unmarried parental status, not living with both parents and having the internet as well as friends as source of sexual health information, were associated with unsatisfactory level of knowledge on sexual health (p<0.05). Age, age at sexual debut and ethnic group of respondents, were not associated with level of knowledge on sexual health (p>0.05).

**Table 1:** sociodemographic characteristics of subjects (N=120)

Variable	Group 1	Group 2	Total	Chi-square
	(Co-education)	(Girl-only)		Statistic
	n=60	n=60	N=120	
	n (%)	n (%)	n (100%)	(p-value)
**Class**				
SSC1	12 (20.0)	10 (16.7)	22 (18.3)	0.61
SSC2	16 (26.7)	19 (31.7)	35 (29.2)	
SSC3	32 (53.3)	31 (51.6)	63 (52.5)	
**Age group (in years)**				
<18	45 (75.0)	48 (80.0)	93 (77.5)	0.51
18-19	15 (25.0)	12 (20.0)	27 (22.5)	
**Age at sexual debut (in years)**				
<14	30 (50.0)	19 (31.7)	49 (40.8)	0.12
15-17	25 (41.7)	35 (58.3)	60 (50.0)	
18-19	5 (8.3)	6 (10.0)	11 (9.2)	
**Parents marital status**				
Married	28 (46.7)	32 (53.3)	60 (50.0)	0.70
Divorced/separated	17 (28.3)	12 (20.0)	29 (24.2)	
Widowed	7 (11.7)	6 (10.0)	13 (10.8)	
Cohabiting	8 (13.3)	10 (16.7)	18(15.0)	
**Ethnic groups**				
Efik	20 (33.3)	12 (20.0)	32 (26.7)	0.10
Ejagham	10 (16.7)	18 (30.0)	28 (23.3)	
Yakurr	5 (8.3)	6 (10.0)	13 (10.8)	
Annang/Ibibio	10 (16.7)	6(10.0)	16 (13.3)	
Ibo	5 (8.3)	12 (20.0)	17 (14.2)	
Others	10 (16.7)	6 (10.0)	16(13.3)	
**Residential status**				
Living with both parents	22 (36.7)	26(43.3)	48 (40.2)	0.49
Living with mother only	14 (23.3)	10 (16.7)	24 (20.0)	
Living with father only	5 (8.3)	2 (3.3)	7 (5.8)	
Living with guardian	19 (31.7)	22 (36.7)	41 (34.2)	
**Source of information on sexual health**				
School teacher	10 (16.7)	13 (21.7)	23 (19.2)	0.01
Other formal teachings	5 (8.3)	19 (31.7)	24 (20.0)	
Family members	6 (10.0)	8 (13.3)	14 (11.7)	
Friends	12(20.0)	6 (10.0)	18 (15.0)	
Internet	24 (40.0)	10 (16.7)	34 (28.3)	
Other informal means	3 (5.0)	4 (6.6)	7 (5.8)	

SSC; senior secondary class

**Table 2:** knowledge and practice of sexual health (N=120)

Variable	Maximum point scale	Group 1 (Co-education)	Group 2 (girl-only)
		n=60	n=60
**All knowledge items**			
Score	62	26.1 ± 7.3	30.4 ± 9.2
T-test p-value			0.01
**Knowledge of anatomical, physiological and psychological puberty changes**			
Score	5	2.7 ± 1.1	3.5 ± 1.2
T-test p-value			0.00
**Knowledge of types of STI**			
Score	10	3.8 ± 2.3	4.1 ± 1.9
T-test p-value			0.44
**Knowledge about transmission of STI**			
Score	15	6.5 ± 3.1	7.4 ± 3.4
T-test p-value			0.13
**Knowledge of symptoms and signs of STI**			
Score	10	3.0 ± 1.8	3.8 ± 2.1
T-test p-value			0.03
**Knowledge of complications of STI**			
Score	6	1.8 ± 0.07	2.9 ± 1.1
T-test p-value			0.00
**Knowledge of prevention of STI**			
Score	11	2.1 ± 1.0	3.7 ± 1.4
T-test p-value			0.00
**Knowledge of unintended pregnancy and its consequences**			
Score	5	2.0 ± 1.2	3.2 ± 1.5
T-test p-value			0.00

STI; sexually transmitted infection

**Table 3:** practice of safe sexual behaviour (N=120)

Practice of safe sexual behavior	Maximum point scale	Group 1 (Co-education)	Group 2 (girl-only)
		n=60	n=60
**Composite score for all items**			
Score	35	20.4 ± 5.1	21.5 ± 4.8
T-test p-value			0.23
**How frequently do you use condom during sex?**			
Score	5	2.7 ± 0.8	2.8 ± 0.7
T-test p-value			0.86
**How frequently do you insist on using condom even when he did not want to use it?**			
Score	5	2.9 ± 0.6	3.0 ± 0.5
T-test p-value			0.74
**How frequently have you had sex with someone that you did not trust or did not test for STI?**			
Score	5	3.2 ± 0.8	2.9 ± 0.7
T-test p-value			0.63
**How frequently have you had sex under the influence of alcohol or other substances?**			
Score	5	2.8 ± 0.7	3.1 ± 0.6
T-test p-value			0.58
**How frequently have you had sex before discussing the risk factors?**			
Score	5	2.8 ± 0.7	2.7 ± 0.8
T-test p-value			0.91
**How frequently do you use emergency contraception pill within 72 hours after having unprotected sex?**			
Score	5	2.8 ± 0.6	2.9 ± 0.5
T-test p-value			0.88
**How often do you watch sexy movies/pictures/magazines?**			
Score	5	2.7 ± 0.8	2.9 ± 0.6
T-test p-value			0.75

**Table 4:** comparison of level of sexual health knowledge between groups

Knowledge category	Group 1 (co-education) n=60	Group 2 (girl-only) n=60	Total n=120	P-value
	n (%)	n (%)	n (%)	
Satisfactory	18 (30.0)	32 (53.3)	50 (41.7)	0.01
Unsatisfactory	42 (70.0)	28 (46.7)	70 (58.3)	

**Table 5:** factors associated with knowledge of sexual health (N=120)

Variable	Knowledge category			
	Unsatisfactory	Satisfactory	Total	P-value
	n=70	n=50	N=120	
	n (%)	n (%)	n (%)	
**Age group (in years)**				
<18	54 (58.1)	39 (41.9)	93 (100)	0.91
18-19	16 (59.3)	11 (40.7)	27 (100)	
**Mean ± SD**	16.3 **±** 1.9	16.4 ± 1.7	16.4 ± 1.8	0.77
**Age at sexual debut (in years)**				
<14	28 (57.1)	25 (41.7)	60 (100)	0.82
15-17	35 (58.3)	25 (41.7)	60 (100)	
18-19	7 (63.6)	4 (36.4)	11 (100)	
**Mean ± SD**	14.2 ± 2.0	14.6 ± 2.4	14.3 ± 2.2	0.32
**Source of sexual health information**				
School teacher	11 (47.8)	12 (52.2)	23 (100)	0.00
Other formal teachings	7 (29.2)	17 (70.8)	24 (100)	
Family members	8 (57.1)	6 (42.9)	14 (100)	
Friends	14 (77.8)	4 (22.2)	18 (100)	
Internet	26 (76.5)	8 (23.5)	34 (100)	
Other informal means	4 (57.1)	3 (42.9)	7 (100)	
**Parent marital status**				
Married	24 (40.0)	36 (60.0)	60 (100)	0.00
Divorced/separated	25 (86.2)	4 (13.8)	29 (100)	
Widowed	9 (69.2)	4 (30.8)	13 (100)	
Cohabiting	12 (66.7)	6 (33.3)	18 (100)	
**Residential status**				
Living with both parents	19 (39.6)	29 (60.4)	48 (100)	0.01
Living with mother only	17 (70.8)	7 (29.2)	24 (100)	
Living with father only	5 (71.4)	2 (28.6)	7 (100)	
Living with guardian	29 (70.3)	12 (29.3)	41 (100)	
**Ethnic groups**				
Efik	18 (56.3)	14 (43.7)	32 (100)	0.98
Ejagham	16 (57.1)	12 (42.9)	28 (100)	
Yakurr	7 (63.6)	4 (36.4)	11 (100)	
Annang/Ibibio	10 (62.5)	6 (37.5)	16 (100)	
Ibo	9 (52.9)	8 (47.1)	17 (100)	
Others	10 (62.5)	6 (37.5)	16 (100)	

## Discussion

The nature of school environment contributes significantly to psychosocial development, as well as sexual health-related (mis)information, beliefs and eventual sexual behavior [18,19]. This is one of few studies in Nigeria, which investigated sexual health knowledge and practice in different school settings characterized by single or coed gender composition of students [20,21]. Key sociodemographic finding was the use of internet as commonest source of sexual health information. The implications of this finding may be far-reaching, considering that adolescents may not be mature enough to appropriately identify credible internet sources, as well as how to scrutinize and utilize web-based sexual health information [28,29]. Misinformation from inappropriate internet sources may contribute to ignorance, misconceptions and poor attitude towards sexuality and safe sexual behavioral practices. However, this finding of internet as prevalent source of information, is at variance with similar study in Ibadan, Oyo State, where internet was least, while school teachers and parents, were the common source of sexual health information [22]. Compared with this study, schools used in the previous study may have had more teachers, that are willing and available for provision of sexual health education to young people. Parents in the previous study, may also have more effective mechanism for restriction of internet use, by their adolescent school children [30]. Key finding in the study was that, compared with coed schools, respondents in girl-only schools had significantly higher level of sexual health knowledge across virtually all constructs, except knowledge of types of STI. Similar study in Ibadan, South West Nigeria, found no significant difference in level of sexual health knowledge comparing coed and single-sex schools [23]. Differences in findings may be due to inclusion of adolescent boys in coed and boys-only schools in the previous study, compared with sole focus on girls and exclusion of boys-only schools in this study. Perhaps inclusion of boys in previous study may have masked assessment of effects of gender-based school environment on adolescent girls as key vulnerable population, within the context of suboptimal sexual and reproductive rights of women in most developing country settings [11,31].

Nevertheless, findings in this study suggests that in the study area or region, compared with coed, girl-only schools may be having more enabling and organized school environmental setting for delivery and reception of sexual health information. Higher level of knowledge on sexual health among respondents in girl-only compared with coed schools, indicates potential role of unique features of peer relationships that are dependent on the school environment. Compared with coed schools, girls in girl-only schools may have more exposure to correct information on sexual health during peer interactions. Generally, girls may be freer and more comfortable with discussing sexual health issues with fellow girls compared with boys. In particular, due to presence of, and occasional or regular interaction with boys, girls in coed schools may have much less sexual health-related interaction especially with their girlfriends, compared with girls in girl-only schools [14,15]. Presence and degree of differences in sex segregation, teacher-student interaction and peer interactions, have been reported to be influenced by gender-based nature of school environment [32]. Such interactions and freedom of expression required for improved sexual health knowledge, is more easily attained in single-sex (girl-only), compared with coed schools. In other words, there may be less potentials for stigma attributable to making inquiries and/or providing apparently sensitive sexual health information in girl-only compared with coed schools. It may therefore be perceived to be easier and more rewarding to conduct formal and informal teachings on sexual health issues in girl-only schools, where group dynamics and interactions are more favorable for better outcomes [32]. This position may be responsible for higher prevalence of school teacher driven and other formal sexual health teachings among girl-only compared with coed schools in this study. Differences in psychosocial perceptions of sexuality, may contribute to higher level of sexual health knowledge in girl-only compared with coed schools. Previous studies among adolescent girls have reported more positive self-concept and academic pursuit, but less traditional orientation to gender roles, among adolescent girls in girl-only compared with coed schools [33]. This background advantage in girl-only schools, may contribute to more enabling environment for their delivery and reception of sexual health information, compared with coed schools [33]. Biological and sociological differences in the way boys and girls learn, may also contribute to variation in levels of sexual health knowledge comparing girl-only and coed schools [34].

Specifically, the effects of these gender-based differences in learning may be more accentuated in single-sex schools, especially considering that opposite-sex interactions in coed schools may diminish the expected differential effects [34]. In other words, in girl-only schools, absence of boys may contribute to optimum utilization of the beneficial learning environment that single-sex school environment provides compared with coed schools. It is also possible that adolescent girls in girl-only schools have less distractions by, and harassment by opposite sex, thereby creating more enabling environment for learning, including sexual health education [35]. In this study, there was no significant difference in sexual behavioral practice scores comparing respondents in coed and girl-only schools. In other words, significantly higher level of sexual health knowledge among respondents in girl-only school, did not translate to similar improvement in sexual health behavioral practices compared with coed school. This finding suggests that, besides level of sexual health knowledge, there are sociocultural, religious beliefs, family life experiences, personality type, peer pressure and other physical developmental factors including pubertal hormonal changes during adolescence, which may be contributing to eventual sexual health practices, in accordance with theory of planned behavior (TPB) [36,37]. Findings from this study, which support potential application of the TPB, include association of poor sexual health knowledge score with use of internet, unmarried status of respondents´ parents, as well as respondents not living with both parents. Similar study in Onitsha, South East Nigeria, also found that not living with both parents was associated with poor sexual knowledge [38]. These findings suggest key role of inappropriate social media exposure, parent-child relationship among other aforementioned constructs, and consideration of views of parents as significant others, in the determination of sexual behavioral practices irrespective of level of knowledge on sexual health. This study has notable limitations that should be considered in the interpretation of the findings. First, lesbianism and bisexual forms of homosexual relationships were not included in this study. Focus on heterosexual practices may have biased report on overall report of safe sexual practices among adolescent school girls in the study setting. Also, presence and nature of parent-child interaction as key determinant of sexual health knowledge and behavioral practice was not assessed [39]. In other words, satisfactory knowledge on sexual health may have been due to adequate exposure to correct information from parents rather than the gender-based nature of the school environment. Yet, findings from this study makes significant contribution to sparse literature on potential role of school environment on sexual health knowledge among adolescent girls in Nigeria.

## Conclusion

There is suboptimal level of sexual health knowledge and practice among in-school adolescent girls in Calabar. Compared with co-educational schools, girl-only schools have better sexual health knowledge, but similar level of behavioral practices. There is urgent need for improvement on sexual health education efforts among adolescent girls, perhaps with more focus on coed schools, within the context of potential inherent disadvantage in the school environmental setting. Also, it may not be out of place to consider hybrid model that incorporates unique features of coed and single-sex school environment. This model may comprise having coed schools, which have the classes segregated by gender, towards retaining potential academic and sexual health benefits of single-sex peer interaction.

### What is known about this topic

- Adolescent girls in developing countries constitute vulnerable subpopulation due to suboptimal implementation of sexual and reproductive health rights and interventions;
- Girl only high school settings are psychosocially different from that of coed schools.

### What this study adds

- Psychosocial differences in the different school settings, includes higher level of sexual health knowledge among adolescent girls in girl-only compared with coed schools;
- Despite significant differences in level of knowledge, the level of sexual behavioral practices is similar, suggesting presence of other essential factors in the pathway towards sexual intention and eventual behavior.
